# Dual PI3K/mTOR inhibitor BEZ235 exerts extensive antitumor activity in HER2-positive gastric cancer

**DOI:** 10.1186/s12885-015-1900-y

**Published:** 2015-11-11

**Authors:** Yan Zhu, Tiantian Tian, Jianling Zou, Qiwei Wang, Zhongwu Li, Yanyan Li, Xijuan Liu, Bin Dong, Na Li, Jing Gao, Lin Shen

**Affiliations:** 1 Department of Gastrointestinal Oncology, Key laboratory of Carcinogenesis and Translational Research (Ministry of Education/Beijing), Peking University Cancer Hospital and Institute, Fu-Cheng Road 52, Hai-Dian District Beijing, 100142 China; 2 Department of Pathology, Key laboratory of Carcinogenesis and Translational Research (Ministry of Education/Beijing), Peking University Cancer Hospital and Institute, Beijing, China; 3 Central Laboratory, Key laboratory of Carcinogenesis and Translational Research (Ministry of Education/Beijing), Peking University Cancer Hospital and Institute, Beijing, China

**Keywords:** Dual PI3K/mTOR inhibitor, HER2, Trastuzumab, Gastric cancer, PDX

## Abstract

**Background:**

To investigate the *in vitro* and in vivo antitumor activity of dual PI3K/mTOR inhibitor BEZ235 (NVP-BEZ235) in HER2-positive gastric cancer.

**Methods:**

HER2-positive breast cancer cell line (BT474), HER2-positive (NCI-N87 and SNU216), and HER2-negative (MKN45) gastric cancer cell lines were used in this study. Cell viability, cell cycle, and HER2 downstream signaling pathways were analyzed using the MTS assay, flow cytometry, and western blotting, respectively. For the in vivo experiments, HER2-positive gastric cancer patient-derived xenografts were treated with BEZ235 to assess its antitumor activity.

**Results:**

The sensitivity of trastuzumab in BT474 cells was higher than that for NCI-N87 and SNU216 cells, which may be partially attributed to continuously active HER2 downstream signaling pathway. BEZ235 inhibited the proliferation of NCI-N87 and SNU216 cells *in vitro* in a dose-dependent manner by inducing the cell cycle arrest at the G1 phase. BEZ235 demonstrated greater inhibitory effects than trastuzumab, a unique targeted drug, in both the *in vitro* and in vivo set of experiments. Additionally, our results indicate that BEZ235 displayed some synergism with trastuzumab. BEZ235 exhibited its antitumor activity in gastric cancer by inhibiting important HER2 downstream signaling pathways, as indicated by the inhibition of phosphorylated AKT and S6.

**Conclusion:**

The present study has demonstrated, for the first time, the antitumor activity of BEZ235 against HER2-positive gastric cancer in patient-derived xenografts, as well its synergistic interaction with trastuzumab. These important findings can be utilized to facilitate the design of future clinical trials.

**Electronic supplementary material:**

The online version of this article (doi:10.1186/s12885-015-1900-y) contains supplementary material, which is available to authorized users.

## Background

In approximately 15–20 % of gastric cancer cases, gastric cells overexpress human epidermal growth factor receptor 2 (HER2) and/ undergo gene amplification [1]. Trastuzumab, a humanized monoclonal antibody that targets the *HER-2/neu* gene, has been widely used to treat HER2-positive breast cancer and gastric cancer. Treatment with trastuzumab showed significantly improved clinical outcomes in patients; however, HER2-positive gastric cancer patients exhibited reduced sensitivity to trastuzumab than the breast cancer patients [2, 3]. Clinical reports indicate that the objective response rate (ORR) of trastuzumab in HER2-positive gastric cancer was lower than that of HER2-positive breast cancer (about 16 % vs. 26 %) [2, 4]. This suggests that HER2-positive gastric cancer has its own molecular characteristics, and therefore, exploring the mechanism that induces differences in the treatment response may eventually provide new therapeutic strategies.

Numerous potential mechanisms for trastuzumab resistance have been reported, such as alterations in the HER2 structure or surroundings, dysregulation of HER2 downstream signaling effectors, and HER2 interactions with other membrane receptors. Of these, the activation of HER2 downstream signaling pathways PI3K/AKT/mTOR and RAS/RAF/MEK/MAPK significantly contributed to trastuzumab resistance [5, 6]. It has been previously reported that trastuzumab reduced the phosphorylation levels of AKT (p-AKT) and S6 (p-S6) in BT474, a trastuzumab-breast cancer cell line. In contrast, trastuzumab treatment in trastuzumab-resistant cell line BT474-TR had no effects on p-AKT and p-S6, indicating that resistance is associated with a failure to inhibit PI3K/mTOR signaling [7, 8].

The association between trastuzumab treatment and PI3K-AKT-mTOR pathway alterations in gastric cancer has not been widely studied. Hence, the objective of this study was to identify alternations in the HER2 downstream signaling pathways post trastuzumab treatment using both *in vitro* and in vivo techniques. Our results will help explore more strategies for improving trastuzumab sensitivity in HER2-positive gastric cancer.

## Methods

### Cell lines, trastuzumab, and inhibitors

MKN45 and NCI-N87 cell lines were provided by Professor You-yong Lv (Peking University Cancer Hospital and Institute), the BT474 cell line was purchased from Peking Union Medical College, and the SNU216 cell line was obtained from Fudan University Shanghai Cancer Center. All the cell lines were cultured in RPMI 1640 medium (Gibco BRL, MD, USA) supplemented with 10 % fetal bovine serum (Gibco BRL), and incubated in a humidified incubator (37 °C) supplemented with 5 % CO_2_.

Trastuzumab was purchased from Shanghai Roche Pharmaceutical Ltd., whereas BEZ235, Everolimus, and AZD6244 were purchased from Selleck China. For the *in vitro* studies, BEZ235, Everolimus, and AZD6244 were dissolved in dimethyl sulfoxide (DMSO) at a stock concentration of 10 mmol/L and stored at −20 °C until further use. Trastuzumab was dissolved in 0.9 % NaCl at a stock concentration of 20 μg/μL and stored at −80 °C, and BEZ235 was formulated in 0.9 % NaCl as a homogeneous suspension (9 mg/mL) and stored at 4 °C until further use in the in vivo experiments.

### Cell viability assay

Cells were seeded at a density of 2000 cells per well in a 96-well plate and incubated overnight in complete medium. Cells were treated with either trastuzumab, BEZ235, Everolimus, AZD6244 alone, or trastuzumab combined with BEZ235 or Everolimus or AZD6244. After 72 h of incubation, cell viability was determined using the MTS tetrazolium substrate (CellTiter 96 Aqueous One Solution Cell Proliferation Assay, Promega, Madison, WI, USA) following the manufacturer’s instructions. The absorbance was measured at 490 nm using a spectrophotometer. All experiments were repeated three times with at least triplicate readings for each concentration.

### Western blotting analysis

Total protein was extracted from cell pellets using CytoBuster Protein Extraction Reagent (Merck Millipore, Darmstadt, Germany). Protein concentration was measured by using a BCA Protein Assay Kit (Beyotime Biotechnology, Jiangsu, China), and 30 μg of protein from each sample was separated by 12 % SDS-PAGE. After transfer, the nitrocellulose membrane (GE Healthcare, Piscataway, NJ) was incubated with the corresponding primary antibodies at 4 °C overnight and secondary antibodies at room temperature for 1 h (the antibody list is shown in Additional file 1: Table S1). Proteins were visualized using ECL plus Western Blotting Detection Reagents (GE Healthcare).

### Cell cycle assay

After 48 h treatment, the cells were harvested and fixed in cold 70 % ethanol overnight at 4 °C. Cells were stained in the dark with 50 μg/mL propidium iodide (BD Biosciences), and incubated at room temperature for 30 min. Cell cycle analysis was performed by FACS Calibur system (BD Biosciences) and analyzed using the ModFit 3.0 software (BD Biosciences).

### Annexin V apoptosis assay

Cell apoptosis was conducted by staining with Annexin V-Allophycocyanin (APC) and 7-amino-actinomycin (7-AAD) (BD Biosciences, Erembodegem, Belgium) for 15 min at room temperature in the dark, followed by flow cytometric analysis within 1 h (BD Biosciences). Cell apoptosis was analyzed using the WinMDI 2.9 software (BD Biosciences).

### Xenograft models in non-obese diabetic/severe combined immunodeficiency (NOD/SCID) mice

Two different xenograft models were used in the present study. NCI-N87 cells (cell density: 1 × 10^6^) were suspended in 0.1 mL of phosphate-buffered saline (PBS), and injected subcutaneously into the dorsal right flank of 6-week-old NOD/SCID mice (Beijing HFK Bio-Technology Co., LTD, Beijing, China). Two HER2-positive patient-derived xenografts (PDX) were obtained using our previously published report. HER2 expression and the characteristics of these two patients are shown in Additional file 1: Figure S1 and Table S2, respectively.

When the tumor volume reached approximately 150 mm^3^, the mice were randomly assigned to four groups (5 mice/group): (1) control group (physiological saline, 100 μL daily), (2) trastuzumab group (20 mg/kg body weight, thrice weekly by intraperitoneal injection), (3) BEZ235 group (45 mg/kg body weight, daily by gavage), and (4) combination (BEZ235, 45 mg/kg body weight, daily by gavage, and trastuzumab, 20 mg/kg body weight, thrice weekly by intraperitoneal injection). The animals were treated for 3 weeks. Tumor size and body weight were measured twice weekly, and the tumor volume (V) was calculated using the following formula: V = L × W^2^/2 (L, long diameter of the tumor; W, short diameter of the tumor). All patients signed written informed consent for their samples to be used in the future study. The experimental procedures involving animals were approved by independent ethics committee of Peking University Cancer Hospital and carried out in accordance with the approved guidelines. The study was undertaken in accordance with the ethical standards of the World Medical Association Declaration of Helsinki. Also, the animal work was approved by Peking University Cancer Hospital’s Institutional Animal Care and Use Committee. All animal experimentation followed internationally recognized ARRIVE (Animal Research: Reporting of In Vivo Experiments) guidelines.

### Immunohistochemical staining

After the mice were sacrificed, the xenografts were isolated, and formalin-fixed paraffin embedded (FFPE) tissue blocks were prepared. Immunohistochemical (IHC) staining was performed as described in our previous study [9]. Briefly, after deparaffinization, hydration, retrieval, and endogenous peroxidase treatment, 4-μm-thick FFPE sections were incubated with the corresponding primary antibodies (the dilutions of p-AKT, p-ERK, and p-S6 were 1:100, 1:400, and 1:200, respectively) overnight at 4 °C. The signal was detected following incubation with IgG-HRP polymer (Cat. PV-6000, ZSGB-BIO, Beijing, China) and diaminobenzidine substrate. Each experiment included a negative control. Sections were interpreted by 2 blinded, independent professional pathologists from the Department of Pathology of Peking University Cancer Hospital. As described previously, staining was scored based on the intensity of staining (1, weak; 2, moderate; and 3, strong) and relative number of cells stained (1: 0–10 %; 2: 11–50 %; and 3: 51–100 %). The expression levels were defined as −, +, ++, and +++ based on the median staining score (intensity score plus percentage score).

### Statistical analysis

Statistical analysis was performed using the SPSS 18.0 software. For the *in vitro* studies, the differences between the groups were analyzed using the unpaired two-tailed *t*-test, one-way ANOVA or factorial analysis. In the in vivo studies, the tumor growth amongst different groups was compared using repeated measures ANOVA. *P* <0.05 was considered statistically significant.

## Results

### Trastuzumab sensitivity in HER2-positive cancer cells and its effects on the HER2 signaling pathway

**Fig. 1:**
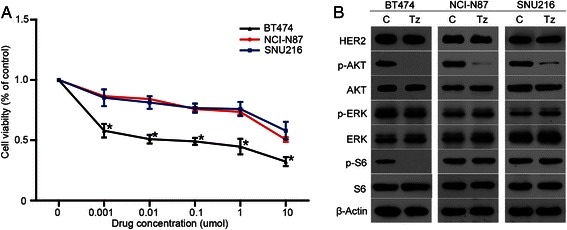
The sensitivity of trastuzumab in HER2-positive cancer cells and its effects on the HER2 signaling pathway. **a** The sensitivity of trastuzumab in HER2-positive breast cancer cells (BT474) was higher than that observed in HER2-positive gastric cancer cells (NCI-N87 and SNU216). **b** Phosphorylated AKT and S6 were both inhibited by trastuzumab in BT474 cells, however, only phosphorylated AKT was inhibited by trastuzumab in NCI-N87 and SNU216 cells. Proliferation assays are expressed as the Mean ± S.D of three replicate assays. Western blots were repeated at least twice. * *P* <0.01 according to factorial analysis. Cropped blots were used because the same experimental conditions were used for running all the gels

The antiproliferative effects of trastuzumab were assessed in HER2-positive breast cancer cell BT474, and gastric cancer cell lines NCI-N87 and SNU216 using a 10-dimensional drug response assay. Consistent with the clinical studies, the sensitivity of trastuzumab in BT474 was higher than that in NCI-N87 and SNU216 (Fig. 1a).

We further evaluated the phosphorylation status of the key proteins in the HER2 signaling cascade. Treatment of BT474 with 1 μmol/L trastuzumab caused almost complete dephosphorylation of both AKT and S6. In contrast, trastuzumab treatment inhibited the phosphorylation of AKT, with no effects on the levels of p-S6 in NCI-N87 and SNU216 cells, indicating that the activity of HER2 downstream signaling pathway influenced the sensitivity of trastuzumab (Fig. 1b).

### BEZ235 strongly inhibits the proliferation of HER2-postive gastric cancer cells in a dose-dependent manner

**Fig. 2:**
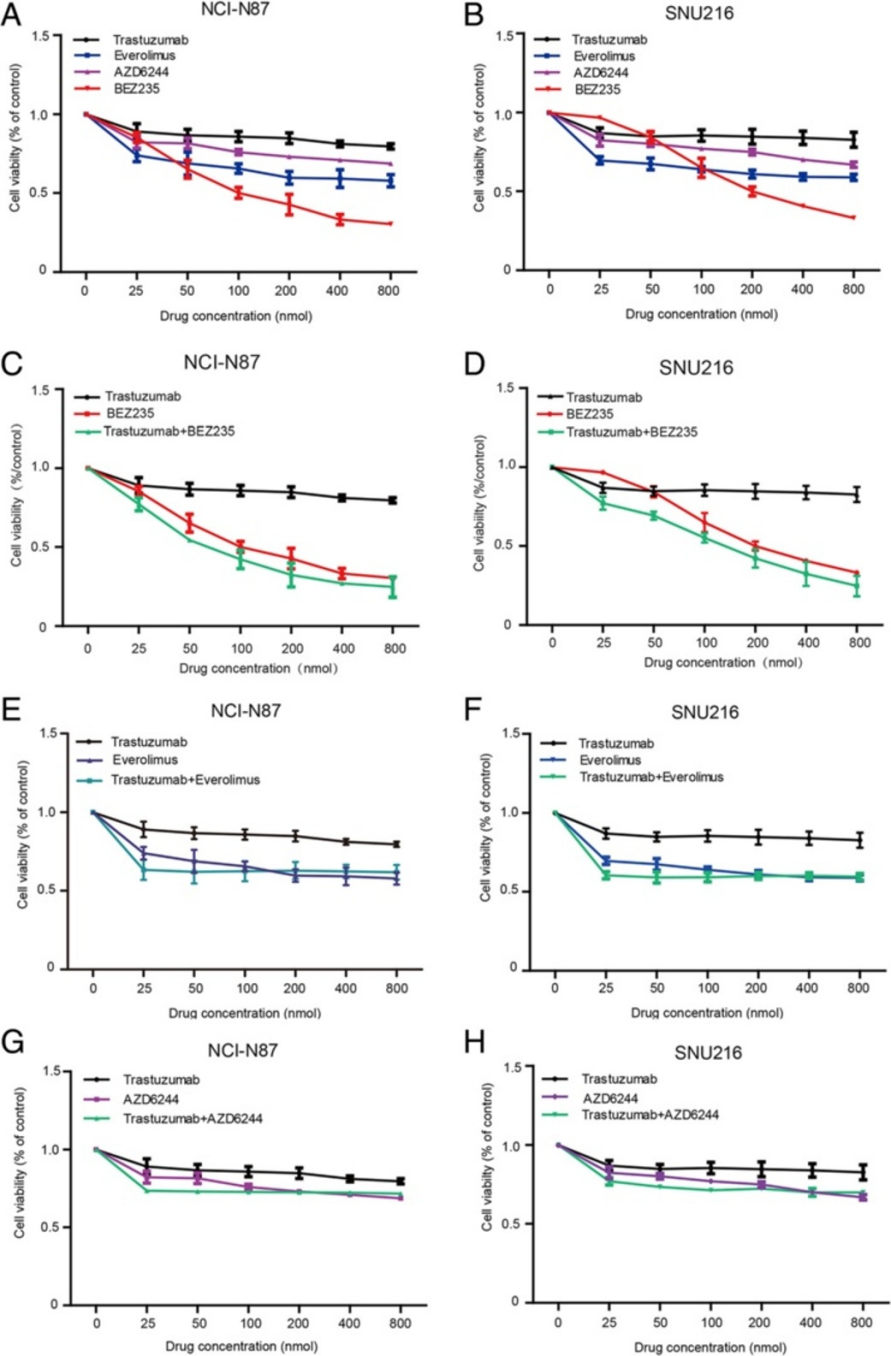
Antiproliferative activity of inhibitors in HER2-postive gastric cancer cell lines. BEZ235 showed superior inhibitory activity against NCI-N87 and SNU216 cells compared to trastuzumab, Everolimus, or AZD6244 (**a**–**b**). Moreover, the synergism was only observed in the combination of trastuzumab with BEZ235 (*P* = 0.000 for all) (**c** and **d**), and not in the combination of trastuzumab with Everolimus or AZD6244 (**e**–**h**). Proliferation assays are expressed as the Mean ± S.D of three replicate assays. *P* values calculated by factorial analysis

To further elucidate the effects of trastuzumab on the HER2 downstream signaling pathways, 3 signaling inhibitors — the dual PI3K/mTOR inhibitor BEZ235, mTOR inhibitor Everolimus, and ERK inhibitor AZD6244 — alone or in combination with trastuzumab, were examined *in vitro*. When used alone, trastuzumab, BEZ235, Everolimus, and AZD6244 inhibited the proliferation of NCI-N87 and SNU216 in a dose-dependent manner. However, compared to trastuzumab, Everolimus, and AZD6244, PI3K/mTOR dual inhibitor BEZ235 exhibited maximum inhibition in the proliferation of NCI-N87 and SNU216 cells (Fig. 2a and b).

Currently, trastuzumab is the only first-line target drug for the treatment of advanced gastric cancer. We, therefore, analyzed the*in vitro* synergistic effects of trastuzumab with BEZ235, Everolimus, or AZD6244. The results indicated that trastuzumab showed maybe some synergism with BEZ235 (all *P* = 0.000) (Fig. 2c and d), but no synergism with AZD6244 or Everolimus (Fig. 2e–h).

### BEZ235 specifically targets HER2-postive gastric cancer cell lines

**Fig. 3:**
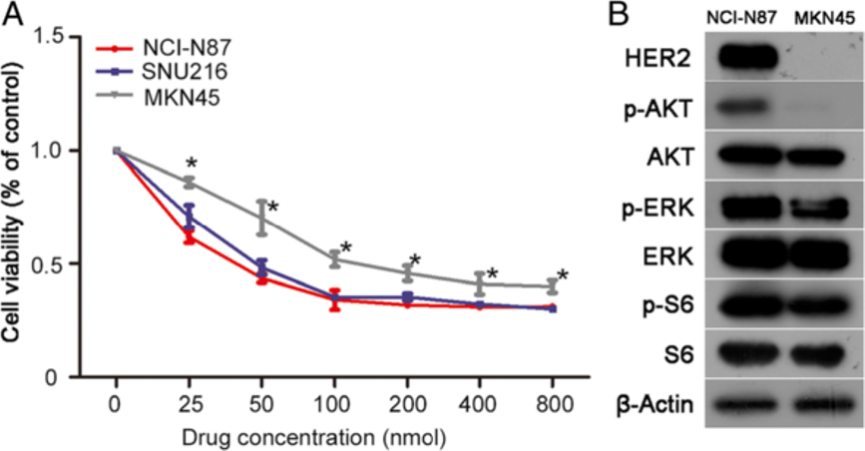
BEZ235 specifically targets HER2-postive gastric cancer cell lines. **a** The sensitivity of BEZ235 in NCI-N87 and SNU216 cells was significantly higher than that observed in MKN45 cells. **b** Western blotting analysis showed that the expression of p-AKT, p-ERK, and p-S6 in MKN45 cells was significantly lower than that observed in NCI-N87 cells. Proliferation assays are expressed as the Mean ± S.D of three replicate assays. Western blots were repeated twice. *P <0.01 according to factorial analysis. Cropped blots were used because the same experimental conditions were used for running all the gels

To confirm the specific activity of BEZ235 treatment on HER2-positive gastric cancer, the antitumor activity of BEZ235 was compared in HER2-positive (NCI-N87 and SNU216) and HER2-negative (MKN45) gastric cancer cell lines. The results indicated that the sensitivity of BEZ235 in NCI-N87 and SNU216 was significantly higher than that observed in MKN45 (*P* = 0.000) (Fig. 3a). Also, the expression levels of p-AKT, p-ERK, and p-S6 in MKN45 cells were significantly lower than that of NCI-N87 cells (Fig. 3b).

### BEZ235 significantly inhibited the HER2 downstream signaling pathways

**Fig. 4:**
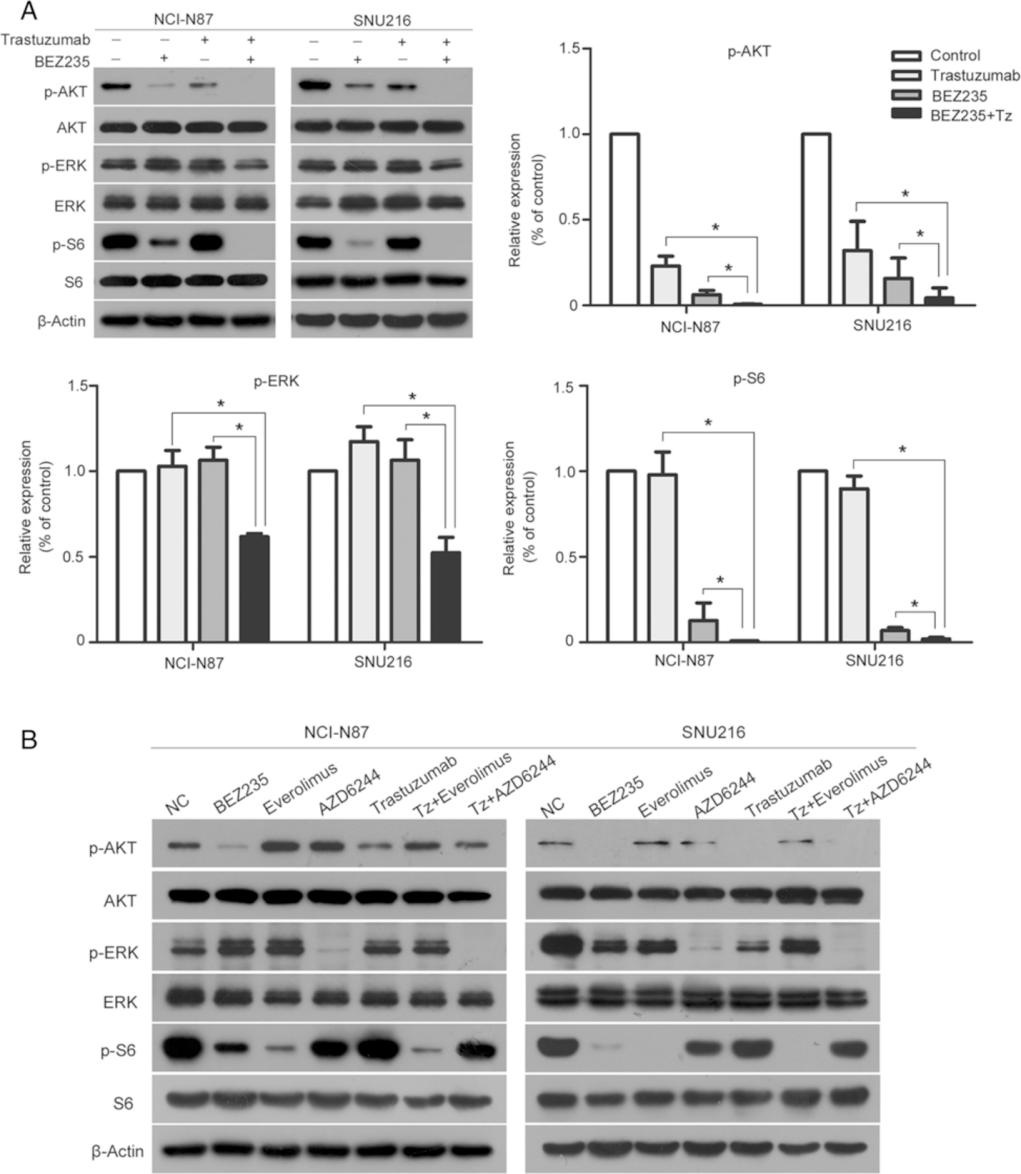
Downregulation of the HER2 signaling pathway *in vitro*. **a** Treatment with BEZ235 in combination with trastuzumab led to complete inhibition of p-AKT and p-S6, with partial inhibition of p-ERK. **b** p-ERK, p-AKT, and p-S6 were not further inhibited by trastuzumab combined with AZD6244 or Everolimus compared to trastuzumab, AZD6244, and Everolimus monotherapy. The levels of p-AKT, p-ERK, and p-S6 were analyzed by western blotting using specific monoclonal antibodies. β-Actin was used as a loading control. The phosphorylation levels of AKT, ERK, and S6 were quantified by using the software Image J. The data are expressed as the Mean ± S.D of three independent experiments. *P <0.01 by one-way ANOVA or unpaired two-tailed *t*-test. Cropped blots were used because the same experimental conditions were used for running all the gels

Immunodetection assay for several HER2 downstream signaling pathway molecules were conducted based on the pervious observation that BEZ235 exerts antiproliferative activity on gastric cancer cells. BEZ235 inhibited the levels of both p-AKT and p-S6, whereas, trastuzumab inhibited only p-AKT in NCI-N87 and SNU216 cells. Interestingly, treatment of NCI-N87 and SNU216 cells with a combination of BEZ235 and trastuzumab completely suppressed the levels of p-AKT and p-S6, with partial inhibition of p-ERK (Fig. 4a). Additionally, treatment with either BEZ235 or trastuzumab did not change the levels of p-ERK, but AZD6244 treatment did reduce p-ERK levels with no changes in p-AKT and p-S6. Everolimus resulted in complete dephosphorylation of S6, with a concomitant increase in the phosphorylation of AKT. Also, p-ERK, p-AKT, and p-S6 were not further inhibited by trastuzumab combined with AZD6244 or Everolimus compared to trastuzumab, AZD6244, or Everolimus alone (Fig. 4b). Since BEZ235 alone significantly inhibited the cell proliferation, PI3K/AKT/mTOR signaling pathway may be more crucial for HER2-positive gastric cancer survival.

### BEZ235 induced cell cycle arrest at the G1 phase

**Fig. 5:**
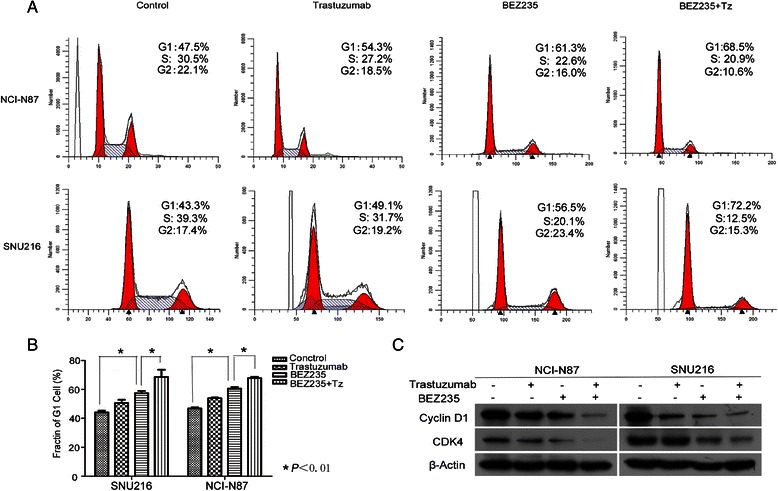
BEZ235 alone or combined with trastuzumab induced cell cycle arrest at G1 phase. **a**–**b** Flow cytometric analysis showed that the percentage of cells accumulated in G1 phase significantly increased after treatment with trastuzumab alone (54.3 and 49.1 %, respectively), BEZ235 alone (61.3 and 56.5 %, respectively), or BEZ235 combined with trastuzumab (68.5 and 72.2 %, respectively), compared to the control (47.5 and 43.3 %, respectively). **c** The expression of CDK4 and cyclin D1 was further downregulated by BEZ235 combined with trastuzumab compared to either one inhibitor used individually. The data are expressed as the Mean ± S.D of three independent experiments. *P <0.05 by one-way ANOVA or unpaired two-tailed *t*-test. Cropped blots were used because the same experimental conditions were used for running all the gels

Cell cycle and cell apoptosis analyses were conducted to explore the potential mechanisms responsible for the inhibition of cell proliferation in gastric cancer cells. The percentage of NCI-N87 and SNU216 cells that accumulated in the G1 phase significantly increased after treatment with trastuzumab alone (54.3 and 49.1 %, respectively), BEZ235 alone (61.3 and 56.5 %, respectively), or BEZ235 combined with trastuzumab (68.5 and 72.2 %, respectively) compared to the control (47.5 and 43.3 %, respectively) (Fig. 5a and b). Concomitant with the cell cycle arrest at the G1 phase, the expression of cyclin-dependent kinase (CDK4) and cyclin D1 were downregulated (Fig. 5c). However, cell apoptosis was not induced by treatment with either BEZ235 or trastuzumab (Additional file 1: Figure S2).

### BEZ235 suppressed the growth of xenografts in vivo

**Fig. 6:**
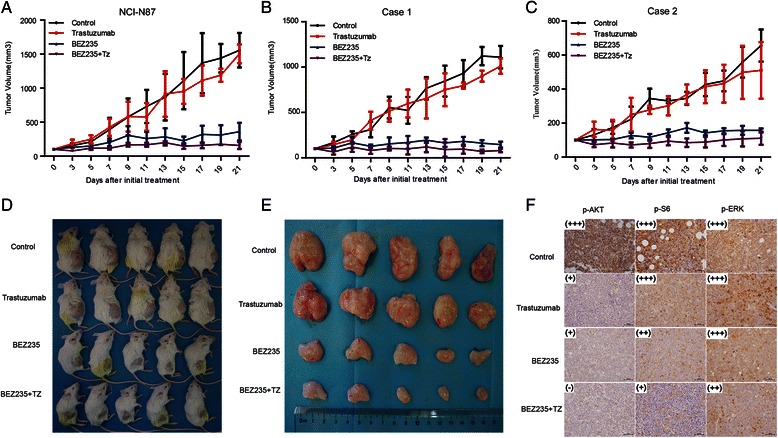
Antitumor effects of trastuzumab and BEZ235 on HER2-positive gastric cancer patient-derived xenografts (PDX). The growth of tumors treated with BEZ235 alone or combined with trastuzumab was significantly suppressed both in the NCI-N87 xenografts (**a**) and in 2 patient-derived xenografts (**b** and **c**). BEZ235 combined with trastuzumab imparted synergistically inhibitory effects on the in vivo xenografts (**a**–**e**). Tumor volumes are expressed as Mean ± S.D. Immunohistochemistry analysis showed that p-AKT, p-S6, and p-ERK were effectively inhibited by BEZ235 combined with trastuzumab compared to BEZ235 alone or trastuzumab alone (**f**). The scale bar represents 100 μm. *P* <0.05 by according to repeated measures ANOVA

NCI-N87 cells and biopsies from gastric cancer patients were subcutaneously inoculated into NOD/SCID mice to establish NCI-N87 xenografts and PDX in vivo. The growth of tumors treated with BEZ235 alone was significantly suppressed both in NCI-N87 xenografts and patient-derived xenografts (*P* = 0.000 for all) compared to the control or the trastuzumab group. Treatment with BEZ235 combined with trastuzumab showed some synergistic inhibitory effects on the in vivo xenografts,but the contribution due to the synergism was relatively modest (*P* value was 0.014 for NCI-N87 xenograft; for two different PDXs, *P* value were 0.036 and 0.03) compared to BEZ235 treatment alone (Fig. 6).

The *in vitro* results that p-AKT and p-S6 could be significantly suppressed by BEZ235 in gastric cancer cell lines were also validated in vivo. Tumor tissues from treated mice xenografts were immunostained with p-AKT, p-S6, and p-ERK antibodies. The results indicated that BEZ235 effectively suppressed p-AKT and p-S6 (Fig. 6f), which is consistent with the results presented in Fig. 4. This suggests that the in vivo efficacy of BEZ235 correlates with PI3K/mTOR target regulation.

## Discussion

Trastuzumab has been widely used for the treatment of HER2-positive breast cancer and gastric cancer, and is currently the only first-line target drug for advanced gastric cancer [2]. However, the lower ORR of trastuzumab in HER2-positive gastric cancer than that of HER2-positive breast cancer prompted us to investigate the potential mechanisms that could provide novel strategies for trastuzumab therapy in HER2-positive gastric cancer.

Our results suggest that the continuously active HER2 downstream signaling pathways might be one of the mechanisms resulting in relatively lower sensitivity of trastuzumab in HER2-positive gastric cancer than breast cancer (Fig. 1). Based on these findings, three signaling inhibitors — the dual PI3K/mTOR inhibitor BEZ235, mTOR inhibitor Everolimus, and ERK inhibitor AZD6244 — targeting the HER2 downstream pathways were screened. Compared to the other inhibitors, BEZ235 showed therapeutic potential in clinical practice (Fig. 2). BEZ235 specifically inhibited the PI3K/mTOR axis and exerted notable antiproliferative activity, indicating that PI3K/mTOR signaling pathway may be more crucial for HER2-positive gastric cancer survival, and that intervention of this pathway may offer better therapeutic options.

Preclinical studies have shown that the dual PI3K/mTOR inhibitor BEZ235 demonstrated antitumor activity in pancreatic cancer and glioblastoma, amongst others [10–12]. Fuereder et al. reported that BEZ235 inhibited the growth of NCI-N87, but not MKN45 and MKN28 xenografts [13], suggesting that BEZ235 might play a specific role in HER2-positive gastric cancer. To validate this finding, a comparative analysis of BEZ235 sensitivity was conducted. The results from this analysis demonstrated that the sensitivity of HER2-positive cells (NCI-N87 and SNU216) was significantly higher than that of the HER2-negative cells (MKN45) (*P* = 0.000) (Fig. 3a). Also, the expression levels of p-AKT, p-ERK and p-S6 in MKN45 cells were significantly lower than the NCI-N87 cells (Fig. 3b), which further validated the fundamental finding of BEZ235 differential sensitivity.

Several preclinical studies have examined the antitumor activity of BEZ235 [8, 12, 14], and the promising results from these have prompted clinicians to assess its efficacy and safety in Phase I/II clinical trials (www.clinicaltrials.gov). O’Brien et al. reported that BEZ235 could overcome the resistance to HER2-targeted therapy in HER2-positive breast cancer in both *in vitro* and in vivo studies [7]. BEZ235 can reverse PI3K/mTOR pathway hyperactivation caused by the oncogenic mutations, and also inhibit HER2-amplified BT474-TR cell proliferation. In the present study, we explored the efficacy of BEZ235 in NCI-N87 and SNU216 trastuzumab-sensitive cells. Therefore, whether BEZ235 could overcome trastuzumab resistance in trastuzumab-resistant HER2-positive gastric cancer cells still remains uncertain. Further studies will be conducted to investigate this using *in vitro* and in vivo trastuzumab-resistant PDX models.

Cell cycle arrest and cell apoptosis are common mechanisms responsible for the inhibition of cell growth. Fuereder et al. [13] reported that the *in vitro* antiproliferative activity of BEZ235 correlated with a dose-dependent increase of gastric cancer cells in the G1 phase of the cell cycle and cyclin D1 downregulation. We confirmed these findings by demonstrating that treatment with BEZ235 alone or in combination with trastuzumab, effectively arrested the cell cycle at the G1 phase, concurrent with the downregulation of CDK4 and cyclin D1 (Fig. 5). Several investigators have reported a controversial role of apoptosis on the antiproliferative activity of BEZ235. Brachmann et al. reported that BEZ235 could induce cell apoptosis in HER2-positive breast cancer cells [15], but in our present study, BEZ235 treatment failed to induce apoptosis in NCI-N87 and SNU216 cells (Additional file 1: Figure S2). Based on our results, we concluded that BEZ235 could not induce a significant apoptotic response, and that the cell cycle arrest at the G1 phase predominantly affects gastric cancer cell proliferation. However, we aim to further explore this mechanism in our future studies.

The GRANITE-1 study indicated that patients with advanced gastric cancer do not benefit from Everolimus [16], although the mechanism behind this observation is unclear. In this study, the results demonstrated that Everolimus treatment inhibited p-S6, but led to increased activation of p-AKT (Fig. 4b). These results might be helpful in understanding the failure of Everolimus in preventing cell proliferation in gastric cancer.

As shown in Fig. 4a, trastuzumab or BEZ235 treatment alone failed to inhibit p-ERK, however, in combination, these two drugs could partially inhibit p-ERK. One of the reasons that we observed this effect could be due to a crosstalk between PI3K/AKT/mTOR and RAS/RAF/MEK/MAPK pathways [17–20]. PI3K can activate RAS through the recruitment of Gab/Shp2 by PIP3 [17], followed by ERK activation. When the PI3K/AKT/mTOR pathway was suppressed by BEZ235 combined with trastuzumab, we observed a decrease in the levels of p-ERK following RAS inhibition. This might explain why BEZ235 had some synergistically inhibitory effects with trastuzumab; however, the synergistic effect was not obvious. Also, BEZ235 and trastuzumab are known to target the PI3K/mTOR pathway. The results also indicate that the ERK signaling pathway might not be important in HER2-positive gastric cancer survival.

Several new drugs such as cetuximab [21], bevacizumab [22], and Everolimus [16] that initially demonstrated antitumor activity in preclinical studies have failed the clinical trials for the treatment of gastric cancer. The most important reason for this negative outcome could be a lack of optimal preclinical animal models. In the present study, patient-derived xenografts models in addition to traditional animal models were used, to confirm the reliability of the results. The results from this study provide useful data to conduct clinical studies for BEZ235 in the future.

## Conclusions

To our knowledge, this is the first report that describes a strong inhibitory activity of dual PI3K/mTOR inhibitor BEZ235 in HER2-positive *in vitro* and in vivo gastric cancer patient-derived xenografts, and this finding can be utilized to design future clinical trials.

## Additional file

Additional file 1: Figure S1. The expression of HER2 in two HER2-positive patient-derived xenografts (PDXs). Cases 1 and 2 with HER2 positive expression of primary tumors maintained positive expression of xenografts based on IHC and DISH results. Scale bars, 100 μm. **Figure S2.** BEZ235 or trastuzumab did not induce cell apoptosis. Flow cytometry analysis showed that the percentage of apoptotic cells did not increased after treatment with trastuzumab alone (4.2 and 4.3 %, respectively), BEZ235 alone (3.0 and 3.2 %, respectively), compared to the control (4.0 and 5.2 %, respectively). The data are expressed as the mean ± s.d of three independent experiments. **P >*0.05 by one-way ANOVA or unpaired two-tailed *t*-test. **Table S1.** Antibodies used in this study. **Table S2.** The characteristics of two patients. (DOC 2229 kb)

## Abbreviations

HER2: Human epidermal growth factor receptor 2
PDX: Patient-derived xenograft
ORR: Objective response rate
p-AKT: Phosphorylated AKT
p-S6: Phosphorylated S6
p-EKR: Phosphorylated ERK
DMSO: Dissolved in dimethyl sulfoxide
7-AAD: 7-amino-actinomycin
NOD/SCID: Non-obese diabetic/severe combined immunodeficiency
PBS: Phosphate-buffered saline
FFPE: Formalin-fixed paraffin embedded
IHC: Immunohistochemistry
CDK4: Cyclin-dependent kinase 4
