# Supplementary material for: Dual PI3K/mTOR inhibitor BEZ235 exerts extensive antitumor activity in HER2-positive gastric cancer
Source: BMC Cancer. 2015 Nov 11;15:894. doi: 10.1186/s12885-015-1900-y (PMC4641417; doi:10.1186/s12885-015-1900-y)
Supplement: Additional file 1: Figure S1. — The expression of HER2 in two HER2-positive patient-derived xenografts (PDXs). Cases 1 and 2 with HER2 positive expression of primary tumors maintained positive expression of xenografts based on IHC and DISH results. Scale bars, 100 μm. Figure S2. BEZ235 or trastuzumab did not induce cell apoptosis. Flow cytometry analysis showed that the percentage of apoptotic cells did not increased after treatment with trastuzumab alone (4.2 and 4.3 %, respectively), BEZ235 alone (3.0 and 3.2 %, respectively), compared to the control (4.0 and 5.2 %, respectively). The data are expressed as the mean ± s.d of three independent experiments. *P >0.05 by one-way ANOVA or unpaired two-tailed t-test. Table S1. Antibodies used in this study. Table S2. The characteristics of two patients. (DOC 2229 kb) [file 12885_2015_1900_MOESM1_ESM.doc]

**Dual** **PI3K/mTOR Inhibitor BEZ235 Exerts Extensive Antitumor Activity in** **HER2-positive Gastric Cancer**

Yan Zhu^*,1^, Tiantian Tian^*,1^, Jianling Zou^1^, Qiwei Wang^1^, Zhongwu Li^2^, Yanyan Li^1^, Xijuan Liu^3^, Bin Dong^2^, Na Li^1^, Jing Gao^#,1^, Lin Shen^#,1^

^1^Department of Gastrointestinal Oncology, ^2^Department of pathology, ^3^Central Laboratory, Key laboratory of Carcinogenesis and Translational Research (Ministry of Education/Beijing), Peking University Cancer Hospital and Institute, Beijing, China

*****These authors contributed equally to this study.

**Correspondence to:**

Professor Lin Shen, Fu-Cheng Road 52, Hai-Dian District, Beijing 100142, China. Tel: +86-10-88196561; Fax: +86-10-88196561; Email: [lin100@medmail.com.cn](mailto:lin100@medmail.com.cn); or Professor Jing Gao, Fu-Cheng Road 52, Hai-Dian District, Beijing 100142, China. Tel: +86-10-88196747; Email: [gaojing_pumc@163.com](mailto:gaojing_pumc@163.com)

**Contributor Information:**

Yan Zhu, Email: zhuyan0398@163.com.

Tiantian Tian, Email: klshangtian@163.com.

Jianling Zou, Email: janely02@126.com.

Qiwei Wang, Email: wangqiweimomo@163.com.

Zhongwu Li, Email: zhwuli@hotmail.com.

Yanyan Li, Email: qiuyesiyu3@sina.com.

Xijuan Liu, Email: liuxj-2003@163.com.

Bin Dong, Email: dongbin@163.com

Na Li, Email: 1500921245@qq.com

Jing Gao, Email: [gaojing_pumc@163.com](mailto:gaojing_pumc@163.com).

Lin Shen, Email: [lin100@medmail.com.cn](mailto:lin100@medmail.com.cn).

**
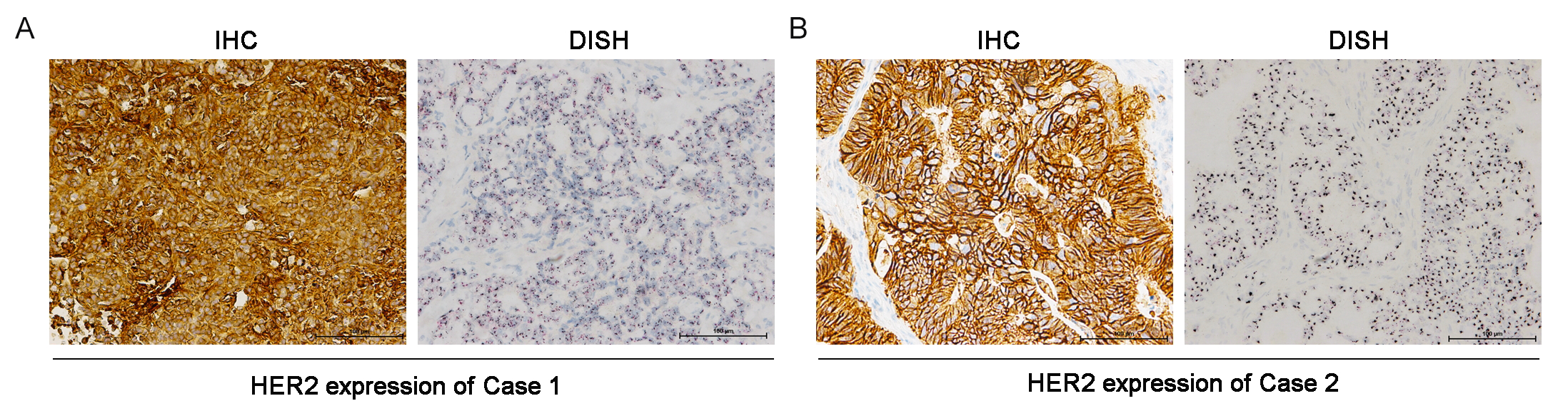
**

**Supplementary Figure S1.** **The expression of HER2 in two HER2-positive patient-derived xenografts (PDXs).** Cases 1 and 2 with HER2 positive expression of primary tumors maintained positive expression of xenografts based on IHC and DISH results. Scale bars, 100μm.


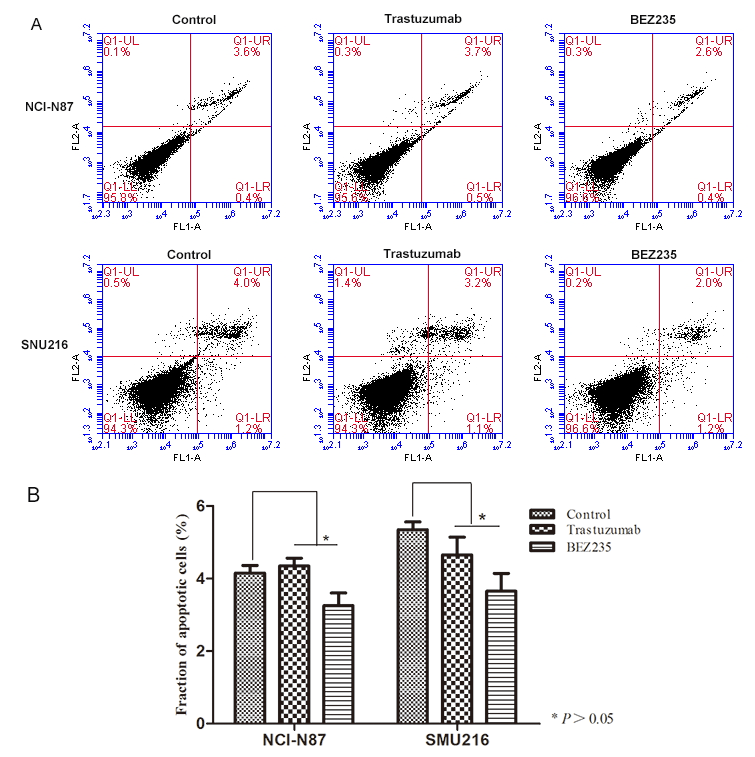


**Supplementary Figure S2. BEZ235 or trastuzumab did not induce cell apoptosis.** Flow cytometry analysis showed that the percentage of apoptotic cells did not increased after treatment with trastuzumab alone (4.2% and 4.3%, respectively), BEZ235 alone (3.0% and 3.2%, respectively), compared to the control (4.0% and 5.2%, respectively). The data are expressed as the mean±s.d of three independent experiments. **P＞* 0.05 by one-way ANOVA or unpaired two-tailed *t*-test.

**Supplementary Table S1. Antibodies used in this study**

| **Antibody** | **Company** | **Catalog number** | **Dilution** | **Source** |
| --- | --- | --- | --- | --- |
| HER2 | Cell Signaling Technology | 4290 | 1:1,000 | Rabbit |
| AKT | Cell Signaling Technology | 9272 | 1:3,000 | Rabbit |
| p-AKT | Cell Signaling Technology | 4060 | 1:1,000 | Rabbit |
| ERK | Cell Signaling Technology | 4695 | 1:3,000 | Rabbit |
| p-ERK | Cell Signaling Technology | 4370 | 1:1,000 | Rabbit |
| S6 | Cell Signaling Technology | 2217 | 1:3,000 | Rabbit |
| p-S6 | Cell Signaling Technology | 4858 | 1:1,000 | Rabbit |
| CDK4 | Cell Signaling Technology | 12790 | 1:1,000 | Rabbit |
| cyclin D1 | Cell Signaling Technology | 2978 | 1:1,000 | Rabbit |
| β-Actin | SIGMA | 122M4782 | 1:5,000 | Mouse |

**Supplementary Table S2. The characteristics** of two patients

| Case | Tumor site | Stage | Differentiation | Lauren classification | HER2 expression |
| --- | --- | --- | --- | --- | --- |
| 1 | Gastric angle | IV | Poor | Diffuse | Positive |
| 2 | Pylorus | IV | Moderate | Intestinal | Positive |
